# Three new species of *Omalodes* (*Omalodes*) (Histeridae, Histerinae) from South America

**DOI:** 10.3897/zookeys.335.5767

**Published:** 2013-09-25

**Authors:** Daniel P. Moura, Lúcia M. Almeida

**Affiliations:** 1 Laboratório de Sistemática e Bioecologia de Coleoptera, Departamento de Zoologia, Universidade Federal do Paraná, Caixa Postal 19020, 81531-980, Curitiba, Paraná, Brazil

**Keywords:** Omalodini, South America, holotype, taxonomy

## Abstract

Three new species of *Omalodes* are described from South America, including descriptions of male and female genitalia. *Omalodes (Omalodes) mazuri* **sp. n.** is described from Peru and Bolivia; *Omalodes (Omalodes) punctulatus* **sp. n.** from Brazil, and *Omalodes (Omalodes) rivus* **sp. n.** from Bolivia and Brazil. This is the first paper with detailed information on the male and female genitalia of the genus, showing a great degree of variation to characterize different species. All three new species can be easily distinguished from other known *Omalodes* and from each other, on the basis of morphology and male genitalia pattern.

## Introduction

The name *Omalodes* was first proposed in the Dejean Catalogue (1833), where 10 species are merely listed. Only a year later those species were properly described by Erichson (1834). Not until 1853 and the following years were new species described for the genus, mainly by Marseul in his “Essai Monographique sur la Famille des Histérides”. In 1861, the genus already had around 35 species, and over the next few years others were described by multiple authors. In 1919, Desbordes described the last three known species of the genus.

When Kryzhanovskij (1972) proposed the tribe Omalodini, the genus had 60 species, divided into 3 subgenera, with *Omalodes* *sensu stricto* beingthe largest, with 51 species. Given the fact that *Omalodes* represent close to 70% of the entire tribe (Mazur 2011), a bigger, more complete study of the genus was important. A complete revision for the subgenus *Omalodes* is being done, and in this manuscript a few of the new species found for this subgenus are described. These species present either a unique combination of characters or are closely related to other species already described, which are broadly distributed across South America. Considering the morphology and distribution of the new species we hope to facilitate their identification.

The subgenus *Omalodes* is characterized by a marginal pronotal stria interrupted behind the head, third dorsal stria of elytra usually weak, present mostly on the anterior half, fourth and fifth dorsal striae usually absent, marginal mesoventral stria in most species widely interrupted, present only at the angles, and lateral metaventral stria continuous with recurrent stria.

Adults of *Omalodes* are collected mostly in decaying trees or other vegetal material, especially when fermenting. Some species can also be found in animal material such as rotten fish, lizards or other animals (personal observation). The genus has a neotropical distribution, with only three species being registered for the southern border of the United States, mostly Florida and Arizona, and not being found on the southern half of Argentina and Chile (Mazur 2011, author unpublished data).

## Material and methods

Descriptive terminology follows Wenzel and Dybas (1941), supplemented by Ôhara (1994) and Lackner (2010). The total length is measured from the anterior margin of the pronotum to the posterior margin of the elytra (to exclude preservation variability in head and pygidial extension), and width is taken at the widest point, generally near the elytral humeri.

The following information is provided for each described species, according to the label: collection data: verbatim citation of country, province or state, locality, date of collection, collector’s name; number of specimens; and collection. The data from each specimen are within double quotes (“ ”). Each line on the same label is separated by a (;). Each label is separated by a slash between spaces (/).

The photographs were taken with a Leica DFC500 digital camera, edited using the software Auto-montage PRO Digital imaging system (SYNCROSCOPY) and Leica MZ16 stereomicroscope.

The studied material belongs to the following institutions: Museu de Zoologia da Universidade de São Paulo, São Paulo (MZSP), Museu Nacional do Rio de Janeiro, Rio de Janeiro (MNRJ), Coleção Entomológica Padre Jesus Santiago Moure, Curitiba, Paraná (DZUP), Museu Anchieta, Porto Alegre, Rio Grande do Sul (MAPA), Museu Paraense Emilio Goeldi, Belém, Pará (MPEG), Brazil; Snow Entomological Collection, University of Kansas Natural History Museum, Lawrence, Kansas (SMEK) and Field Museum of Natural History, Chicago, Illinois (FMNH), United States of America. The type material was deposited in the museum from which it was originally borrowed.

## Taxonomy

### Omalodes (Omalodes) mazuri sp. n.

http://zoobank.org/B4C51AD8-179C-4DC0-B44E-FCDD2CD882F4

http://species-id.net/wiki/Omalodes_mazuri

**Figure 1.**
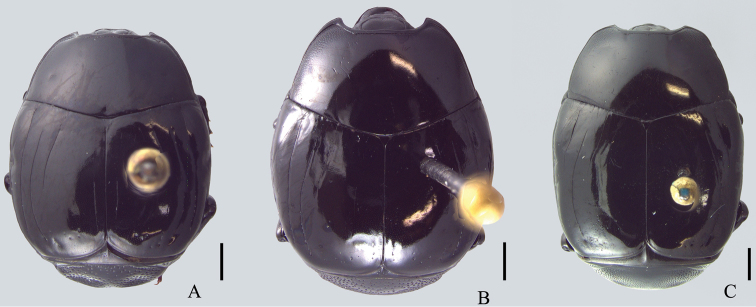
Dorsal view. **A** *Omalodes (Omalodes) mazuri* sp. n. **B** *Omalodes (Omalodes) rivus* sp. n. **C.** *Omalodes (Omalodes) punctulatus* sp. n. Scale: 0.5 mm.

**Figure 2.**
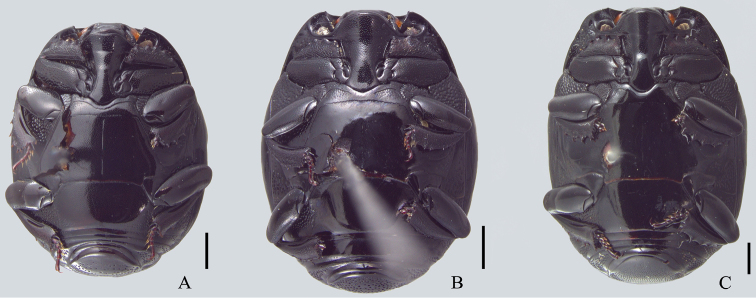
Ventral view. **A** *Omalodes (Omalodes) mazuri* sp. n. **B** *Omalodes (Omalodes) rivus* sp. n. **C.** *Omalodes (Omalodes) punctulatus* sp. n. Scale: 0.5 mm.

**Figure 3.**
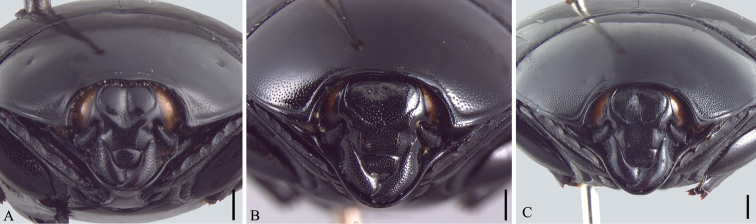
Head and pronotum, frontal view. **A** *Omalodes (Omalodes) mazuri* sp. n. **B** *Omalodes (Omalodes) rivus* sp. n. **C** *Omalodes (Omalodes) punctulatus* sp. n. Scale: 0.25 mm.

**Figure 4.**
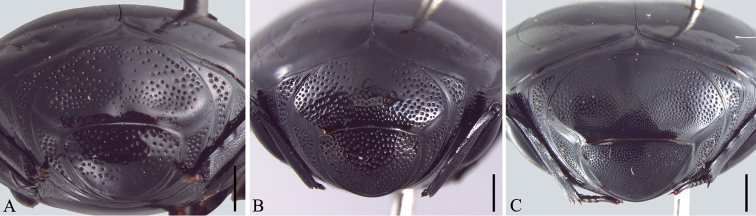
Propygidium and pygidium. **A** *Omalodes (Omalodes) mazuri* sp. n. **B** *Omalodes (Omalodes) rivus* sp. n. **C** *Omalodes (Omalodes) punctulatus* sp. n. Scale: 0.25 mm.

**Figure 5.**
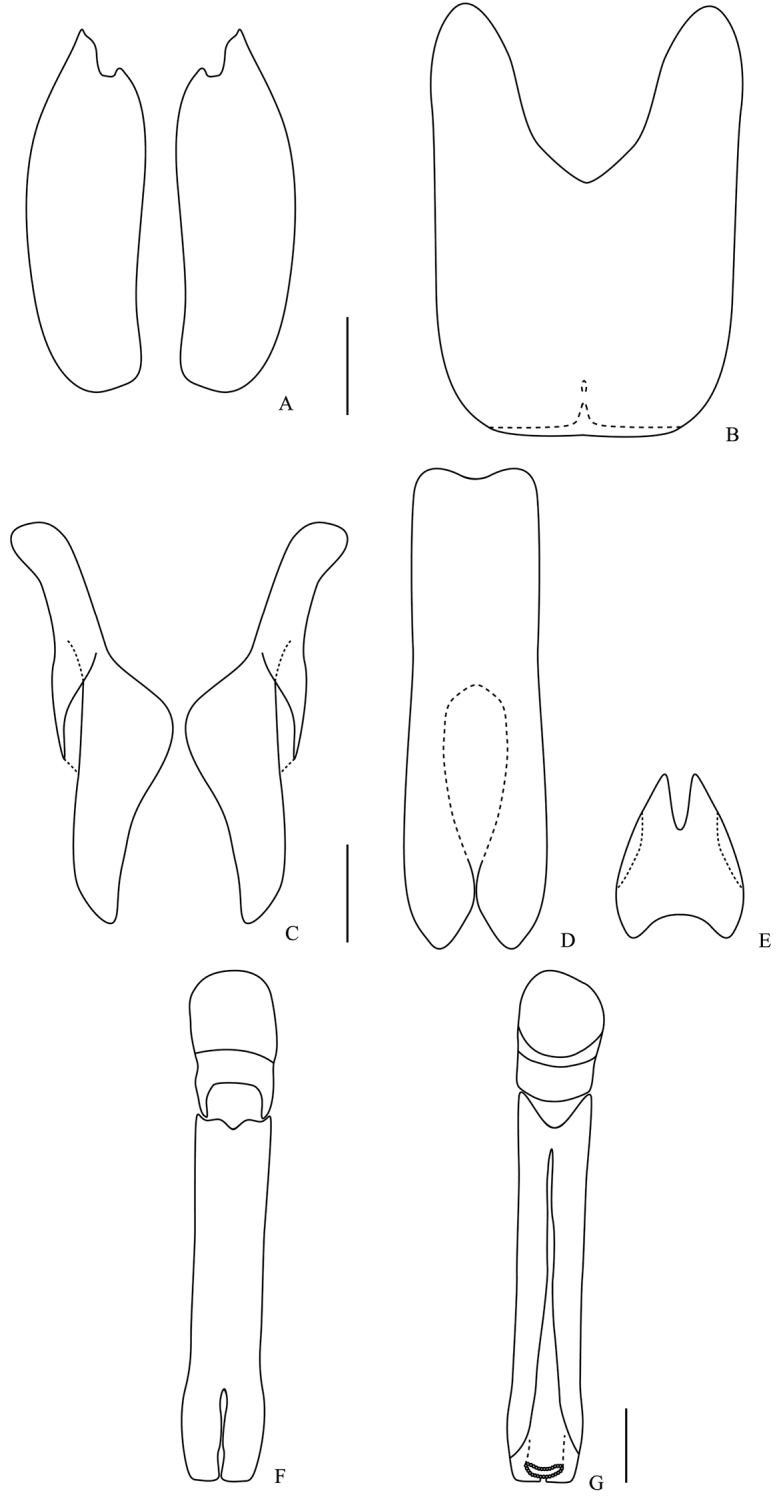
Male genitalia. *Omalodes (Omalodes) mazuri* sp. n. **A** eighth sternite **B** eighth tergite **C** ninth tergite **D** “spiculum gastrale” **E** tenth tergite **F** aedeagus, dorsal view **G** aedeagus, ventral view. Scale: 0.25 mm.

Figs 1A , 2A , 3A , 4A , 5A–G

#### Type material.

**Holotype: Male. BOLIVIA:** “Bolivia tropical; Region Chapare; I:5:50, 400m; R. Zischka leg. / regarded by Mazur as n. sp.” (FMNH). **Paratype: Male. PERU:** “PERU: Cusco, Villa Carmen Field; Stn., ~1.7 km W cafeteria, res.; trns. 12.89250°S, 71.41917°W; 555m. 22–24.v.2011. Flight; intercept. D. J. Bennett & E. Razuri; PER-11-FIT-007” (SMEK).

#### Diagnosis.

Frons with a sulcus, little wider close to the epistoma (Fig. 3A); sutural stria present on posterior half (Fig. 1A); prosternal keel with carinal striae indicated on the posterior half, along the prosternal process; marginal mesoventral stria complete, beginning close to mesometaventral suture and continuous along the anterior and lateral margins (Fig. 2A).

#### Description.

*Size range*: Length: 5–6.5mm, Width: 4–5mm. *Body form* (Fig. 1A): Body oval, convex, piceous, covered with micropunctures. *Head* (Fig. 3A): Frontal stria complete, anterior portion somewhat projected towards epistoma, bordering a broad sulcus, connected to supraorbital stria on each side; labrum subtrapezoidal, almost rectangular, anterior margin straight, posterior margin articulated with epistoma, about 1.5 times as wide as long; mandibles short, without subapical teeth. *Pronotum* (Figs 1A, 3A): Sides rounded, narrower anteriorly, with a pair of foveae close to lateral punctures on posterior half; marginal pronotal stria beginning at anterior angles, interrupted behind head, continuous to posterior margin; lateral pronotal stria present, indicated along lateral and anterior margins; lateral punctures of pronotum indicated close to lateral margin, except for posterior fourth, where absent or indicated by weaker punctures, almost as fine as ground punctures. *Elytra* (Fig. 1A): With depression on each side, posterad to humeral stria; marginal epipleural stria absent; epipleural stria complete; outer subhumeral stria present at posterior third, inner subhumeral stria absent; first dorsal stria just slightly shortened at posterior margin; second dorsal stria absent from anterior fifth; third dorsal stria present, strongly marked behind base, weaker close to anterior margin, with two or three punctures close to posterior margin; sutural stria indicated in posterior half; apical stria absent. *Prosternum* (Fig. 2A): Prosternal lobe rounded, slightly emarginated at middle of anterior margin, marginal stria complete; lateral punctures of prosternal keel present; carinal striae of prosternal keel present only in posterior half; prosternal process rounded. *Mesoventrite* (Fig. 2A): Marginal mesoventral stria complete, continuous along lateral and anterior margins; mesometaventral stria somewhat crenulated medially with mesometaventral suture visible along its length. *Metaventrite* (Fig. 2A): Lateral metaventral stria continuous with recurrent stria. *Abdomen* (Figs 2A, 4A): Abdominal ventrites smooth medially, punctuated on sides, the punctures somewhat irregular; propygidium with punctures more dense and regularly distributed close to posterior half and lateral margins, sparser and more irregular close to anterior margin, with a pair of impressions on the posterior half, on each side; pygidial punctures more or less regularly distributed, although sparse, especially in the middle. *Male genitalia* (Figs 5A–G): Eighth sternite divided in two longitudinally elongate sclerites, base with shallow emargination; eighth tergite subrectangular, with a pair of anterolateral projections, one on each side, apex with small, less sclerotized area along margin, medially curved with small desclerotized point before curvature; “spiculum gastrale” elongate, sclerotized in anterior half, base slightly concave; ninth tergite divided in two elongate sclerites, wider medially, apex without emargination; tenth tergite composed of a single sclerite, widely emarginated at anterior half, somewhat concave at apex, wider at posterior half and with small desclerotized area along lateral margin; aedeagus elongate, cylindrical, basal piece about two times as long as wide, parameres just slightly fused anteriorly on the ventral side, almost completely fused dorsally, truncate at apex; basal margin with a wide “V”-shaped emargination ventrally and superficial one dorsally; parameres a little wider close to the apex and slightly dilated at basal margin in lateral view. *Female Genitalia*: Unknown.

#### Etymology.

This species is named in honor of Dr. Slawomir Mazur, a great researcher of Histeridae.

#### Distribution.

This species is known from Bolivia, Region Chapare and Peru, near Cusco. The holotype was probably collected close to Chapare, Cochabamba.

#### Remarks.

The complete marginal mesosternal stria of this species is somewhat peculiar in regards to the subgenus *Omalodes*. For the already known species, only *Omalodes (Omalodes) fortunatus* Lewis, 1898 has a similar stria. The sutural stria is longer than the usual for the subgenus, being indicated on the posterior half as a well marked, continuous stria. The only species that have the sutural stria as long as this species are *Omalodes (Omalodes) pulvintatus* Erichson, 1834 and *Omalodes (Omalodes) mendax* Marseul, 1861, but both have the stria indicated by a series of punctures. In other species of the subgenus the stria is absent or only indicated by a few punctures close to the posterior margin of the elytra. Even though those characters are a little different from usual for the subgenus, this species can be easily placed inside *Omalodes* *s. str.* when a wider set of characters is taken into account.

The paratype of this species lacks the pair of pronotal foveae close to the lateral punctuation. This probably means that the character is somewhat variable or more probably that those fovea are a developmental anomaly.

### Omalodes (Omalodes) rivus sp. n.

http://zoobank.org/2BCE1143-798F-4569-81BA-0D7956B43D18

http://species-id.net/wiki/Omalodes_rivus

**Figure 6.**
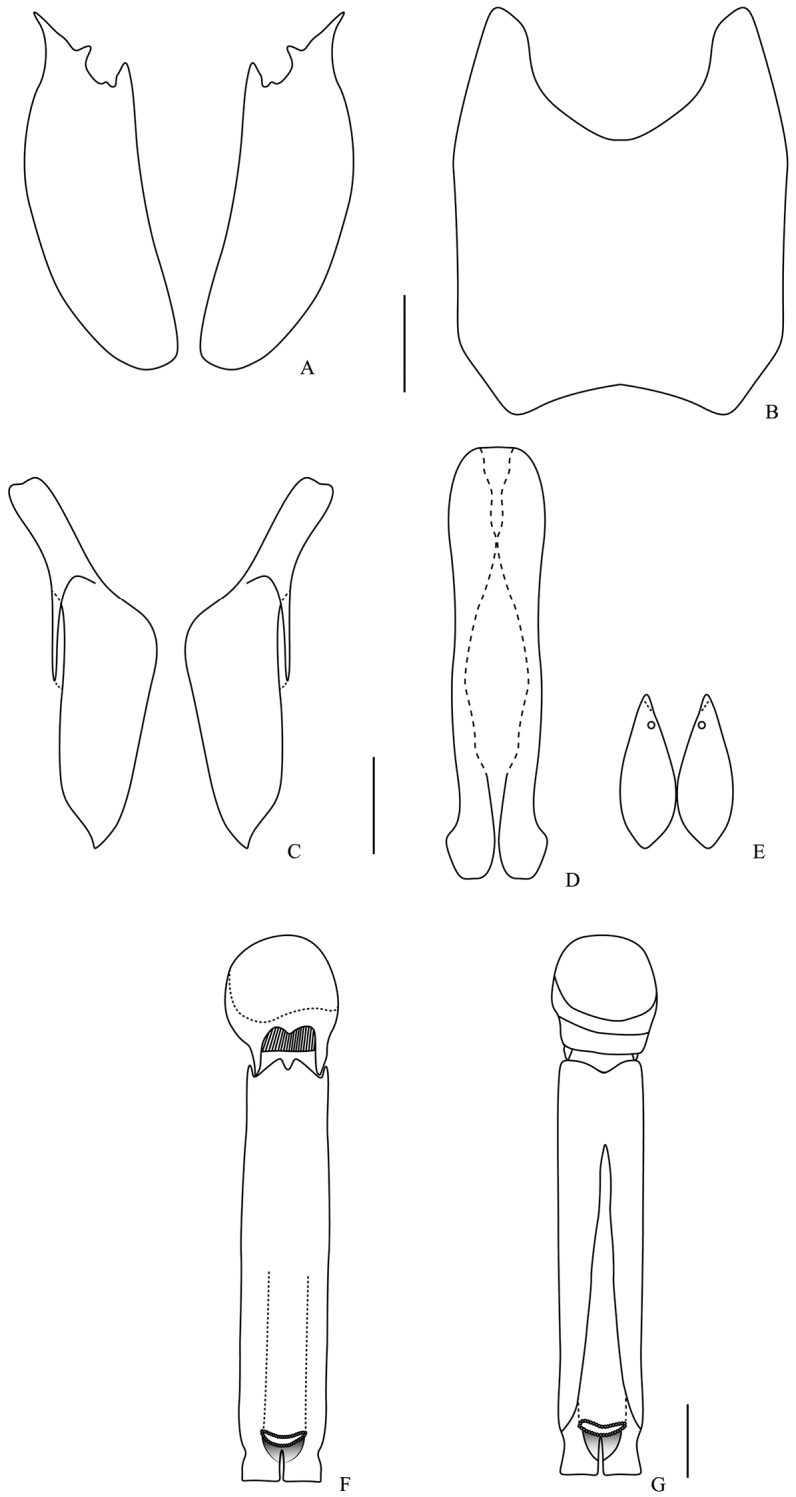
Male genitalia. *Omalodes (Omalodes) rivus* sp. n. **A** eighth sternite **B** eighth tergite **C** ninth tergite **D** “spiculum gastrale” **E** tenth tergite **F** aedeagus, dorsal view **G** aedeagus, ventral view. Scale: 0.25 mm.

Figs 1B , 2B , 3B , 4B , 6A–G , 8A–E

#### Type material.

**Holotype: Male. BRAZIL:**“Brasil Pará; Serra Norte; Manganes M_2_; 13/X/1986 / Armadilha; Shanon / Brasil Pará; J. Dias” (MPEG). **Paratypes: BOLIVIA:**“Bolívia; Pcia Ichilo; Buenavista XI.1950; A. Martinez leg.”, 1 specimen; (MZSP); **BRAZIL:** “Brasil, Amapá; Serra do Navio; X-1996; P. Magno leg. / Assentamento”, 1 specimen (MNRJ); “Coleção; M. Alvarenga / Colônia Rio Branco; Óbidos Pará BRASIL; 5-XII-1952; P. A. Teles col.”, 1 specimen (DZUP); “Coleção Campos Seabra / Mangabeira MOCAJUBA; Pará BRASIL; II-1953; Orlando M. Rego”, 1 specimen (MNRJ); “Coleção Campos Seabra / Óbidos; Pará BRASIL; I-1954 J. Brazilino”, 2 specimens (MNRJ); “Brasil; Santaremsinho; Mun. de Itaituba; Rio Tapajós = Pará; VI-62 Dirings”, 1 specimen (MZSP); “Itaituba, PA; XII.1963”, 1 specimen (MZSP); “Brasil, Pará; Marajó-Breves; Rio Caruacá; 22.II.1988 / Area 1; Margem–SE; Isca 1 Solo”, 5 specimens (MPEG); “Brasil, Pará; Marajó-Breves; Rio Caruacá; 22.II.1988 / Area 1; Isca 2; Coleta solo”, 1 specimen (MPEG); “Brasil, Pará; Marajó-Breves; Rio Caruacá; 22.II.1988 / Area 2; Margem W; Isca 2”, 5 specimens (MPEG); “Brasil, Pará; Marajó-Breves; Rio Caruacá; 22.II.1988 / Area 2; Isca 2; Margem W; Coletas solo”, 1 specimen (MPEG); “Brasil Pará; Marajó-Breves; Rio Caruacá; 4.VIII.1988 / Brasil PA; J. Dias / B55; L17”, 1 specimen (MPEG); “Brasil Pará; Marajó-Breves; Rio Caruacá; 6.VIII.1988 / Isca Laranja / M. Martins”, 1 specimen (MPEG); “Brasil Pará; Marajó-Breves; Rio Caruacá; B60; 6.VIII.1988 / Brasil Pará; J. Dias”, 1 specimen (MPEG); “Brasil Pará; Marajó-Breves; Rio Caruacá; 9.VIII.1988; B-66–Isca Laranja / Brasil Pará; J. Dias”, 2 specimens (MPEG); “Brasil Pará; Marajó-Breves; Rio Caruacá; 12.VIII.1988; B72; Isca abacaxi / Brasil Pará; J. Dias”, 1 specimen (MPEG); “Brasil Pará; B72; Marajó-Breves; Rio Caruacá; 12.VIII.1988; Isca laranja / Brasil Pará; J. Dias”, 1 specimen (MPEG); “Brasil Pará; Serra Norte; Pojuca; 4.VII.1985 / Brasil Pará; R. B. Neto”, 1 specimen (MPEG); “Brasil Pará; Serra Norte; M2 Mata; Isca de fruta; 24 a 26.I.1986 / Brasil Pará; M. F. Torres”, 1 specimen (MPEG); “Brasil Pará; Serra Norte; Manganês M_2_; 13.X.1986 / Armadilha; Shanon / Brasil Pará; J. Dias”, 1 specimens (MPEG); “Brasil Pará; Serra Norte; Manganês; Isca 2.4; 13.X.1986 / Brasil Pará; J. Dias”, 1 specimen (MPEG); “Brasil Pará; Serra Norte; 3 Alfa; 15 a 18.X.1986 / Armadilha Shanon; J. Dias”, 1 specimen (MPEG); “Brasil Pará; Serra Norte; N2; Isca fruta; 22 a 24.I.1986 / Brasil Pará; H. Andrade”, 1 specimen (MPEG); “Brasil Pará; Serra Norte; N2; Isca fruta; 22 a 24.I.1986 / Brasil Pará; M. F. Torres”, 1 specimen (MPEG); “Brasil Pará; Paragominas; Faz. Cachoeira; do Rio Vermelho; 18 a 21.I.1991 / Brasil Pará; P. Tadeu / Armadilha Malayse”, 1 specimen (MPEG); “Brasil Pará; Paragominas; Faz. Cachoeira; do Rio Vermelho; 18.I.1991 / Brasil Pará; P. Tadeu”, 1 specimen (MPEG); “Brasil Pará; Benevides; F. Morelândia; 6.III.1987 / Brasil Pará; J. Dias”, 1 specimen (MPEG); “Coleção Campos Seabra / Guajará; Borba Amazonas; Brasil IV.1943; A. Parko”, 1 specimen (MNRJ); “Brasil: AM; Itacoatiara; V/1962 Dirings”, 1 specimen (MZSP); “Forte Príncipe da; Beira. Rondônia; 19.XI-3.XII.1967; G. R. Kloss col. / *Omalodes* sp.; N. Degallier”, 1 specimen (MZSP).

**Diagnosis:** Frons flat or slightly concave (Fig. 3B); outer subhumeral stria long, present at least on posterior half, usually beginning close to the end of humeral stria; propygidium punctate, little sparser at anterior half (Fig. 4B); marginal mesosternal stria interrupted, present in angles and slightly along anterior margin (Fig. 2B).

#### Description.

*Size range*: Length: 5–6.5mm, Width: 4–5mm. *Body form* (Fig. 1B): Oval, convex, piceous, covered with micropunctures. *Head* (Fig. 3B): Frons flat or with medial longitudinal impression, slightly concave; frontal stria complete or slightly interrupted medially, joined with supraorbital stria laterally at posterior margin; labrum subrectangular with anterior margin nearly straight, posterior margin with a small semicircular smooth area; about two times as wide as long; mandibles short, without subapical teeth. *Pronotum* (Figs 1B, 3B): Sides rounded, narrower anteriorly; marginal pronotal stria beginning at angles, interrupted behind the head, continuous to posterior margin; lateral pronotal stria present, indicated along lateral and anterior margins; lateral punctures not strongly indicated, but easily visible, equally covering lateral margin. *Elytra* (Fig. 1B): Marginal epipleural stria absent, elytra with superficial impression in its place; epipleural stria complete; outer subhumeral stria present at posterior half, beginning close to humeral stria, sometimes slightly interrupted medially or anteriorly; inner subhumeral stria absent; first dorsal stria slightly shortened near posterior margin, indicated in posterior third by a series of punctures; second dorsal stria shortened in anterior margin, indicated in posterior fourth by a series of punctures; third dorsal stria weakly indicated, continuous in anterior half, with some punctures near posterior margin; a few punctures close to the posterior margin; sutural stria variable, usually present in posterior half, posterior third or indicated by series of irregularly spaced and variably intense punctures. *Prosternum* (Fig. 2B): Prosternal lobe rounded, marginal stria complete along anterior margin; lateral punctures of prosternal keel present, weakly indicated; prosternal keel without carinal striae; prosternal process rounded. *Mesoventrite* (Fig. 2B): Marginal mesoventral stria interrupted, present in lateral margin and slightly along anterior margin; mesometaventral stria present, almost straight, slightly curved towards prosternal process. *Metaventrite* (Fig. 2B): Lateral metaventral stria continuous with recurrent stria. *Abdomen* (Fig. 2B): Ventrites smooth medially, with irregular punctures on sides; propygidium almost completely punctate, weaker and more dispersed in middle of anterior half; pygidium punctate regularly distributed. *Male genitalia* (Figs 6A–G): Eighth sternite divided in two longitudinally elongate sclerites, base with irregular emargination; eighth tergite subrectangular, with pair of anterolateral projections, apex concave; “spiculum gastrale” longitudinally elongate and with anterior margin rounded; ninth tergite divided in two longitudinally elongate sclerites, wider medially, apex without emargination; tenth tergite with two sclerotized areas, longitudinally elongate, almost parallel sided, with small anterior non-sclerotized circular area; aedeagus elongate, cylindrical, basal piece widely and deeply emarginated dorsally; parameres almost completely fused dorsally, except for posterior tenth; fused only in anterior third ventrally, basal margin with a couple of projections dorsally and a concave emargination ventrally; apex truncated, with superficial constriction on each side. *Female Genitalia* (Figs 8A–E): Eighth sternite with sides rounded, basal margin irregularly sclerotized, apex slightly curved, proximal baculi not fused to sternites; eighth tergite trapezoidal, larger at base, with non-sclerotized medial area, apex with non-sclerotized area medially and laterally; oval area in coxites covered with dense and strong punctures, with setae laterally and ventrally, gonostyle only visible ventrally, articulated with coxites, with three apical setae, two long and one short, valvifer elongate, enlarged at anterior third; spermatheca elongate, smooth in posterior third, annulated on anterior 2/3; spermathecal gland longer than spermatheca, wider in posterior half.

#### Etymology.

The name of this species refers to its main localities of collection being close to rivers, especially in Pará, Brazil.

#### Distribution.

Pará, Rondônia, Amapá, Amazonas (Brazil) and Ichilo (Bolivia). Mostly of the material was collected close to large rivers of those regions, especially in Pará, along the Caruaca river.

#### Remarks.

This species resembles a few others in the subgenus; the frons and the length of the outer subhumeral stria is quite similar to *Omalodes (Omalodes) gagatinus* Erichson, 1847 and *Omalodes (Omalodes) anthracinus* Marseul, 1854, but this stria is a little longer on *Omalodes (Omalodes) rivus*. The punctures of the propygidium is reminiscent of that in *Omalodes (Omalodes) lucidus* Erichson, 1834, being completely covered by punctures which are sparser and weaker on the anterior half. The ninth tergite is more or less similar to all other species of the subgenus, but in *Omalodes (Omalodes) rivus* it is wider in the posterior half, and has a very subtle emargination along the anterolateral margin, this emargination is not known for any other species in the subgenus.

#### Observations.

There are few specimens with specific ecological data associated. Some labels indicate that the bait was fruit (orange or pineapple), probably rotten, since some other species of the genera are known to be attracted only by rotting vegetal or fruit material.

### Omalodes (Omalodes) punctulatus sp. n.

http://zoobank.org/82F13716-A5D8-4541-AE55-CFA548C49ABF

http://species-id.net/wiki/Omalodes_punctulatus

**Figure 7.**
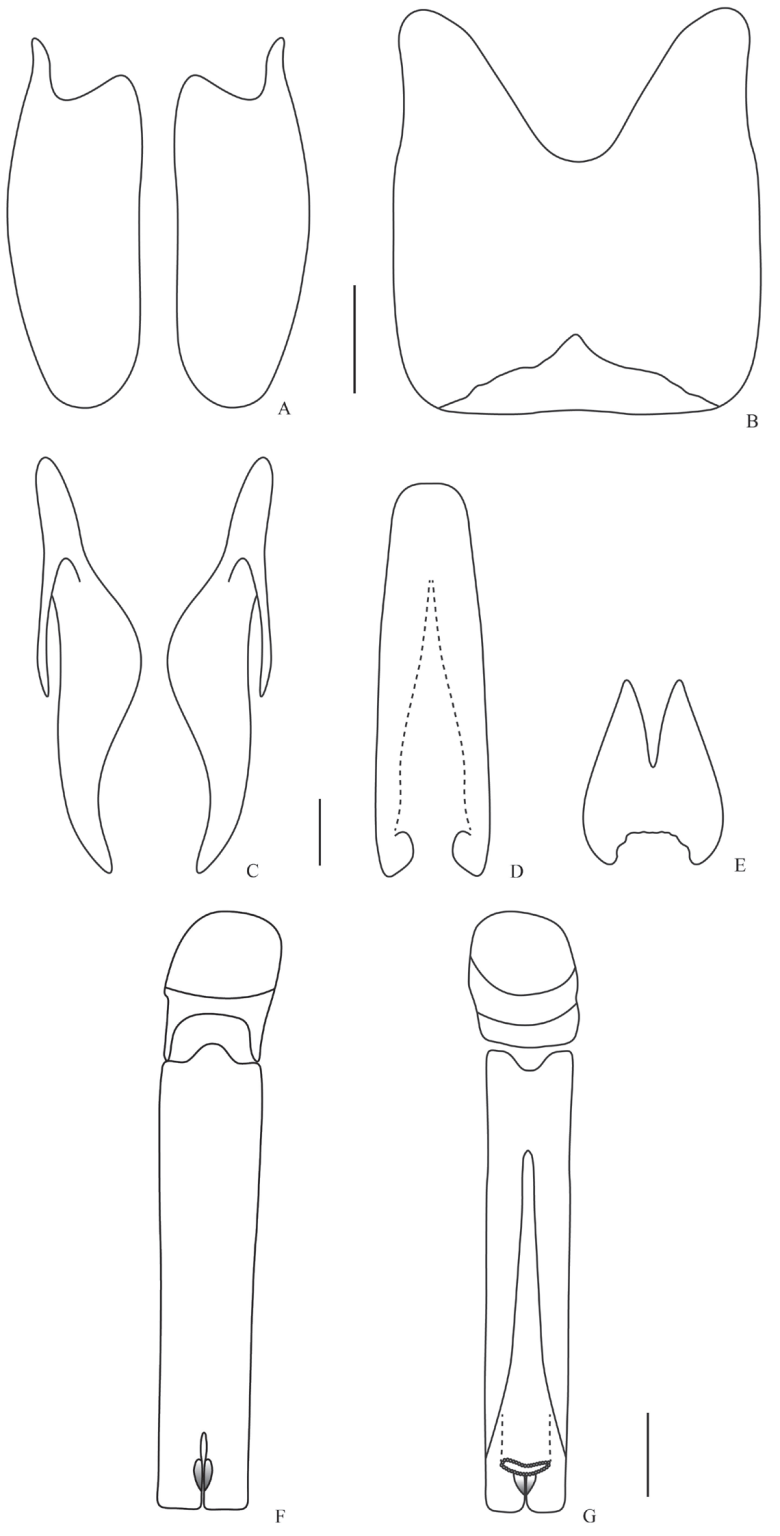
Male genitalia. *Omalodes (Omalodes) punctulatus* sp. n. **A** eighth sternite **B** eighth tergite **C** ninth tergite **D** “spiculum gastrale” **E** tenth tergite **F** aedeagus, dorsal view **G** aedeagus, ventral view. Scale: 0.25 mm.

Figs 1C , 2C , 3C , 4C , 7A–G

#### Type material.

**Holotype: Male. BRAZIL:** “15.IX.1955; Barueri; S. Paulo; 5876 / K. Lenko Leg. (MNRJ) **Paratypes**: **BRAZIL**: “Itapiranga; II 55”, 2 specimens (MAPA); “Itapiranga; II 55”, 2 specimens (DZUP); “Est. Minas; Araguary; 5.XII.1921; Melim”, 1 specimen (MNRJ); “1225; 9.IX.1954; Barueri; Leg. K. Lenko”, 1 specimen (MNRJ); “29.X.1955; Barueri; S. Paulo; 6658 / K. Lenko Leg.”, 2 specimens (MNRJ); “Serro Azul; 3.40”, 1 specimen (MAPA); “1-44; Paraná; Monjolinho / 1628 / Coleção; F. Justus Jor.”, 1 specimen (DZUP); “Pelotas; II 55”, 1 specimen (DZUP).

#### Diagnosis.

Frons punctate with a medial fovea (Fig. 3C); dorsal elytral striae weakly indicated (Fig. 1C); propygidium almost completely covered with punctures, except for a semicircular area on anterior margin and with a pair of superficial impressions, one on each side at posterior half (Fig. 4C).

#### Description.

*Size range*: Length: 7–8mm, Width: 5–6mm. *Body form* (Fig. 1C): Oval, convex, piceous, covered with micropunctures. *Head* (Fig. 3C): Frons impressed medially, with a well delimited fovea, punctate; frontal stria complete, slightly curved close to antennal insertion, continuous to epistoma, curved reaching the medial fovea, joined with supraorbital stria laterally at posterior margin; labrum subrectangular with anterior margin nearly straight, about two and a half times as wide as long; mandibles short, without subapical teeth. *Pronotum* (Figs 1C, 3C): Sides rounded, narrower anteriorly; marginal pronotal stria beginning at angles, interrupted behind the head, continuous to posterior margin; lateral pronotal stria present, indicated along lateral and anterior margin, slightly shortened near posterior margin; lateral punctures covering entire lateral margin, more visible and covering a wider area at anterior third. *Elytra* (Fig. 1C): Marginal epipleural stria absent; epipleural stria complete; outer subhumeral stria present at posterior third; inner subhumeral stria present, indicated by few weak punctures beginning at posterior third and continuous at posterior fifth; all dorsal striae weakly indicated, first stria slightly interrupted at anterior margin, with a curvature near humerus and two weak punctures near posterior margin; second dorsal stria beginning a little after the anterior margin, continuous until and indicated by two stronger wider punctures at posterior margin, third dorsal stria weakly indicated, continuous at anterior half, with some punctures near posterior margin; fourth and fifth dorsal striae absent; few punctures close to posterior margin; sutural stria indicated by few weak punctures at posterior margin. *Prosternum* (Fig. 2C): Prosternal lobe rounded, marginal stria complete along anterior margin; lateral punctures of prosternal keel present; prosternal keel without carinal striae; prosternal process rounded. *Mesoventrite* (Fig. 2C): Marginal mesoventral stria only present at angles; mesometaventral stria absent, mesometaventral suture visible and almost straight. *Metaventrite* (Fig. 2C): Lateral metaventral stria continuous with recurrent stria. *Abdomen* (Fig. 2C): Ventrites smooth medially, punctures somewhat irregular on the sides; propygidium with pair of impressions on posterior half and almost completely covered with strong punctures laterally, weaker towards the middle and a smooth semi-circular area on anterior margin; pygidium almost completely covered with punctures except for a small smooth area along lateral and posterior margin. *Male genitalia* (Figs 7A–G): Eighth sternite divided in two longitudinally elongate sclerites, base with a wide and regular emargination; eighth tergite subrectangular, with pair of anterolateral projections, a more sclerotized triangular area at apex; “spiculum gastrale” longitudinally elongate; ninth tergite divided in two longitudinally elongate sclerites, wider medially, apex without emargination; tenth tergite wider at posterior half, with deep emargination in anterior half and a wider, irregular one at apex; aedeagus elongate, cylindrical, parameres almost completely fused dorsally, except for posterior fifth, fused in anterior fifth ventrally, basal margin with medial emargination ventrally and projected dorsally in the middle; apex truncate. *Female genitalia*: The female genitalia of this species resemble that of *Omalodes (Omalodes) rivus*, but spermatheca and spermathecal gland were not prepared for examination in this species.

**Figure 8.**
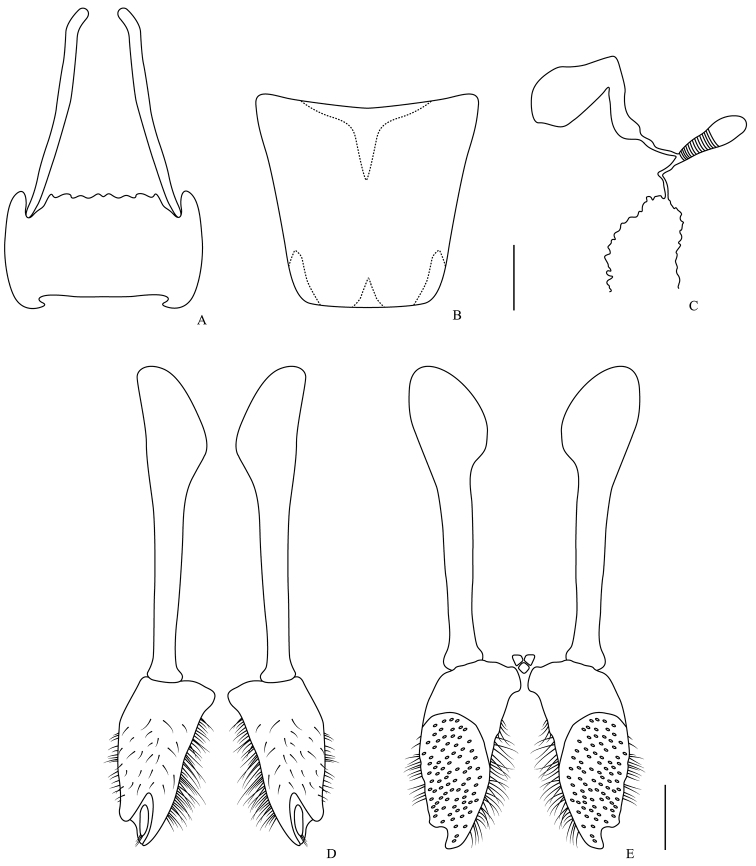
Female genitalia. *Omalodes (Omalodes) rivus* sp. n. **A** eighth sternite and proximal baculi **B** eighth tergite **C** spermatheca and spermathecal gland **D** gonostyle, coxites and valvifer, ventral view **E** coxites and valvifer, dorsal view. Scale: 0.25 mm.

#### Etymology.

The name of this species refers to the punctures of the pronotum, which are peculiar for the subgenus.

#### Distribution.

The known distribution for the species is the southern and southeastern region of Brazil, ranging from Araguari, Minas Gerais; going through Barueri, São Paulo; Cerro Azul and the Monjolinho Natural Reserve, Paraná; Itapiranga, Santa Catarina until Pelotas, Rio Grande do Sul.

#### Remarks.

*Omalodes (Omalodes) punctulatus* has a few punctures near the posterior margin of the elytra, and because of this, appears closely related to *Omalodes (Omalodes) lucidus*. However, its punctures are less numerous and more regular (maybe related to the 4th and 5th dorsal striae), while the punctures of *Omalodes (Omalodes) lucidus* are more numerous and irregularly distributed over the posterior margin of the elytra. Likewise the dorsal striae are weakly indicated, as in *Omalodes (Omalodes) gagatinus* and *Omalodes (Omalodes) anthracinus*, but when compared to those species, its striae are not so weakly indicated nor highly interrupted or shortened. The punctures of the propygidium resembles that of *Omalodes (Omalodes) planifrons* but it differs from this species by the punctures in the pronotum and its frons with a medial fovea. When compared to *Omalodes (Omalodes) rivus* this species has a shorter outer subhumeral stria, present only at posterior third and the frons has a medial fovea while in *Omalodes (Omalodes) rivus* the outer subhumeral stria is present in the posterior half and the frons is plan or only slightly concave.

The inner subhumeral stria can be present only at the posterior fifth or absent; the punctures next to the posterior margin of the elytra can vary in number and intensity; the sutural stria can be completely absent and a few specimens have the dorsal striae more strongly indicated, although never as strongly as some other species of the genus.

## Supplementary Material

XML Treatment for Omalodes (Omalodes) mazuri

XML Treatment for Omalodes (Omalodes) rivus

XML Treatment for Omalodes (Omalodes) punctulatus
